# Evaluating non-cognitive skills in medical school applicants

**DOI:** 10.1186/s12909-024-05046-5

**Published:** 2024-01-23

**Authors:** Katya Peri, Mark J. Eisenberg

**Affiliations:** 1 Lady Davis Institute for Medical Research, Jewish General Hospital, Montreal, QC Canada; 2 Departments of Medicine and of Epidemiology, Biostatistics and Occupational Health, McGill University, Montreal, QC Canada; 3 Division of Cardiology, Jewish General Hospital, McGill University, 3755 Côte Ste-Catherine Road, Suite H-421.1, H3T 1E2 Montreal, QC Canada

## Abstract

Medical school candidates must have both the cognitive and professional competencies required to become good physicians. In this commentary, we outline the evidence and outcomes associated with the implementation of these selection methodologies and evaluate their ability to assess non-cognitive skills.

## Introduction

Deciding which candidates to admit to medical school is a daunting task. Applicants must have the cognitive ability to complete the rigorous medical school curriculum and the professional qualities necessary to provide quality medical care [1]. Every year, the number of medical school applicants is well above the number of available seats, with an average of 5.5% of applicants being accepted into the incoming cohort [2]. Thus, the need for an accurate selection process is clear. Currently the Medical College Admissions Test (MCAT) and Grade Point Average (GPA) are used to assess candidates’ academic achievement [3]. However, these examinations cannot predict whether an applicant possesses the critical thinking leadership and judgement skills required to become a good physician [3]. Existing methods of evaluating personal characteristics, namely personal statements, reference letters and essays, have proven inadequate at predicting personal characteristics [4]. A selection tool that is easily accessible and measures personal/professional qualities is required in the medical school application process. The Multiple Mini Interviews (MMIs) and the Computer-based Assessment for Sampling Personal characteristics (CASPer) were introduced in order to fill this gap [5]. In this commentary, we outline the evidence and outcomes associated with the implementation of these selection methodologies.

### Multiple Mini Interviews (MMIs)

McMaster University pioneered the MMI in 2004 to address two problems; (i) traditional interviews did not accurately determine medical school performance and (ii) patient complaints related progressively more to physicians’ non cognitive skills such as judgement, communication and ethics [6]. Unlike traditional panel interviews, the MMIs consist of 6–10 timed scenarios that students complete individually. Each scenario aims to evaluate a competency required in the medical field, such as leadership, empathy, communication, and collaboration [7]. The scenarios can present students with an ethical dilemma, a conflict they must resolve, a riddle they must interpret or a graphic they must describe to an evaluator will rate their performance. Students are marked separately by two evaluators who rank their performance on a scale of 1–5, one being unacceptable and five being excellent [8]. The candidate’s scores are used to determine admission to the medical program [9].

Several studies have evaluated the MMI with regards to professional outcomes. Strengths of the MMI include reliability, predictive validity, feasibility, and practicality [1, 10, 11]. This study also found that the MMI is a flexible tool that can measure ethics, morals, communication skills, problem solving and critical thinking [6]. The interview structure remained reliable across various education settings employed in 11 different countries. When administered virtually, the MMI retains its reliability [5]. The MMI can also somewhat identify a candidate’s argument ability, reasoning skills and situational examination skills. It also gives the committee an idea of the candidate’s interpersonal skills, thoughtfulness, and demeanor in a short period of time [12]. Importantly, MMI scores were correlated with future performance on the OSCE (Objective Structured Clinical Examination), national council examinations and licensing exams [13]. The weakness of this exam lies in its inability to evaluate emotional intelligence or personality traits [14]. Very little investigation as to the use of the MMI as a professional skills assessment tool exists. Additional research evaluating this purpose must be conducted before the MMI can become a permanent fixture of the medical school application.

### The CASper exam

The CASPer exam was also introduced by McMaster University to screen for professional characteristics before students are invited to the MMIs [15]. The MMI cannot be administered to all applicants, since the technical and staff resources required are too great [16]. The CASPer exam was introduced to serve as an accessible, simple and reliable way to screen applicants for desirable non-cognitive abilities before the MMI [16]. The CASPer’s computer-based nature means it can be applied to the entire application pool as a reliable and valid screening tool [17]. This situational judgement test involves 12 scenarios, eight video-based and four written, where applicants are required to describe how they would respond to certain situations [18]. Like the MMI, these situations can include ethical dilemmas, workplace conflicts or prompts to recount and evaluate personal experiences. The scenarios are timed, and candidates must respond to all questions within the five-minute restriction per situation. The open-ended questions allow applicants to incorporate their unique backgrounds or experiences into their answers and provide context-specific responses [17]. Each question is graded by a single evaluator using a numerical Likert scale from 1 to 9 (one being unsatisfactory and nine being excellent) [18]. The candidate’s total score is then ranked into quartiles with the first quartile representing a score in the bottom quarter and the fourth quartile in the top 75–100% of test takers [18]. The aim of this exam is to allow selection to be based upon the student’s ethics, decision making and professionalism [18]. Like the MMI, the CASPer test has high overall test reliability and inter-rater reliability [19]. Thus, this assessment has potential to be used as a screening exam in conjunction with cognitive assessments to identify qualified candidates for medical school interviews.

Several studies have been conducted to evaluate the CASPer as a selection tool, specifically as a predictor of licensing exam success. One study reports a modest but significant predictive power of the CASPer’s situational judgement questions with scores on Canadian national licensing examinations and case-based panel review three to six years in the future [15, 17]. These results suggest a moderate predictive ability of the CASPer exam to estimate future performance on behavioural tests [15, 19]. Importantly, the CASPer exam’s predictive ability for professional qualities is similar to that of the MCAT and GPA for cognitive abilities [15]. However, like the MMI, it is unknown whether the CASPer exam accurately evaluates professional skills like communication and empathy.

### A holistic approach to medical school admissions

Medical school admissions committees have focused on admitting students who they predict will be successful in the foundational science curriculum of medical education [1]. Heavy emphasis is therefore placed on academic performance in prerequisite science courses as well as on the MCAT [1]. Recently,, medical schools have been transitioning towards a holistic admissions process that evaluates applicants based on their experiences and individual attributes in addition to their grades [20]. These personal characteristics are taken into consideration to identify candidates who would not only thrive as medical students, but also potentially contribute value to the medical community Importantly, holistic review does not abandon assessment of academic achievement, but rather broadens the evaluation process to include the evaluation of non-cognitive skills in the application procedure.

### Potential obstacles with a holistic approach

Many medical schools have removed the MCAT requirement [21]. The MCAT has widely been accepted as a useful way to determine student’s academic ability [1]. Removing this benchmark and favouring students with greater non-cognitive skills raises the question of whether the upcoming cohorts will be well versed enough in the sciences to successfully contribute to the medical field. Superior knowledge of medicine and science is required for physicians to provide quality care and admission committees have the responsibility of selecting candidates that are most likely to succeed in medical school and on their licensing exams [22]. Solely relying on GPA as a measure of academic ability is a risk, considering the wide range of program difficulty, unique university grading criteria and curving of final marks. For instance, an A in one school may translate to a B + at another. The standardization of the MCAT exam addresses these inconsistencies [23]. Six out of 12 medical schools in Canada and ten med schools in the U.S have dropped the MCAT [24]. It will be important to closely monitor upcoming cohorts to document the consequences of these changes on students’ success and patient outcomes.

The correlation between CASPer scores and MMI scores is important to study in order to further refine the medical school admissions process. Should both scores be highly correlated, they could possibly be evaluating similar characteristics and are therefore redundant. If the scores are not correlated, it would be useful to know which professional characteristics each test evaluates. Analysis regarding the correlation between CASPer test scores and MCAT and GPA should also be conducted, as this would provide a thorough and holistic view of the use of each test in the admission process. To date, very little evidence these subjects exists. Admission officers should be using evidence-based methods to determine how to evaluate potential candidates, therefore filling this knowledge gap is of utmost importance.

Furthermore, as the MMI and CASPer have been gradually incorporated into admission processes, increasing numbers of preparatory courses and private coaching for these tests are appearing on the market [25]. These services, like those for the MCAT, are expensive, rendering them inaccessible to students with a lower SES [25]. Therefore, it is possible that in the future, students from more affluent backgrounds will be more successful at the MMI and CASPer [26]. Since the MMI and CASPer are often conducted online, high speed internet access is imperative. Students in underserved areas may experience disruptions, impacting their ability to complete the exam or interview and consequently affect their evaluation [27]. As the admission process continues to change, research examining the sociodemographic effect of these modifications must be undertaken to ascertain its impact on medical schools’ entering classes.

## Conclusion

The need to accurately evaluate both non-cognitive ability and academic prowess in medical school applicants is important to select capable and professional future physicians. The medical school admission process has been undergoing constant reform over the last few decades [28]. Gradually, medical schools have been dropping the autobiographical sketch, personal essay, and panel interview in favour of an individualized and holistic evaluation of candidates [2]. Today, the MCAT and GPA are still used as sufficient measures of cognitive ability and the MMI and CASper appear to be appropriate assessments to evaluate professional qualities. Current research suggests that the MMI and CASPer are feasible and reliable selection tools with moderate predictive validity in evaluating performance on future licensing exams. Currently, 16 US medical schools and 17 Canadian medical schools require CASPer while over 30 US medical schools and 12 Canadian medical schools use the MMI [29–31]. Before solidifying their place in the admission process, admission committees must be sure that these exams are measuring non-cognitive skills. Limited evidence proving this relationship exists. In addition, the correlation between each test score must be evaluated to determine redundancy and specify evaluated characteristics. Moreover, the gradual shift towards MMI and CASPer and away from MCAT and GPA raises questions regarding future cohorts’ academic ability. Will this transition negatively impact students’ success or quality of care? There exists little research involving the evaluation of medical schools’ metrics with the aim of quality improvement in the admissions process. This information is invaluable to direct future reform. Future research should also include regression-based studies of medical student performance and the health of their patients to ascertain the impact of these changes.

## Data Availability

Not applicable.
